# Dissecting Metabolic Functions and Sugar Transporters Using Genome and Transportome of Probiotic *Limosilactobacillus fermentum* KUB-D18

**DOI:** 10.3390/genes16030348

**Published:** 2025-03-17

**Authors:** Yuke He, Kevin Mok, Pramote Chumnanpuen, Massalin Nakphaichit, Wanwipa Vongsangnak

**Affiliations:** 1 Interdisciplinary Graduate Program in Bioscience, Faculty of Science, Kasetsart University, Bangkok 10900, Thailand; yuke970710@gmail.com; 2 Department of Biotechnology, Faculty of Agro-Industry, Kasetsart University, Bangkok 10900, Thailand; kevin.m@ku.th; 3 Center of Excellence for Microbiota Innovation, Faculty of Agro-Industry, Kasetsart University, Bangkok 10900, Thailand; 4 Department of Zoology, Faculty of Science, Kasetsart University, Bangkok 10900, Thailand; pramote.c@ku.th; 5 Omics Center for Agriculture, Bioresources, Food and Health, Kasetsart University (OmiKU), Bangkok 10900, Thailand

**Keywords:** *Limosilactobacillus fermentum* KUB-D18, bioinformatics, probiotics, genome, transportome

## Abstract

**Background/Objectives:** *Limosilactobacillus fermentum* KUB-D18, a heterofermentative lactic acid bacterium with promising probiotic properties, is known for promoting gut health and nutrient absorption. Originally isolated from chicken intestines, this strain demonstrates versatile metabolic capabilities in diverse gastrointestinal environments. However, the metabolic functions and sugar transport-related genes remain largely unexplored. This study thus aimed to dissect metabolic functions and sugar transports of *L. fermentum* KUB-D18. **Methods:** Next-generation and third-generation sequencing techniques using integrative genomic platform towards transportome analysis were performed. **Results:** The complete genome, sized at 2.12 Mbps with a GC content of 51.36%, revealed 2079 protein-encoding genes, of which 1876 protein functions were annotated and identified in top categories involved in amino acids, nucleotide, energy, and carbohydrate transports and metabolisms. Comparative genes analysis identified 50 core and 12 strain-specific genes linked to probiotic properties, e.g., acid resistances and bile tolerances, antioxidant functions, or anti-inflammatory properties. Further, sugar transportome analysis uncovered 57 transporter genes, demonstrating diverse carbon utilization and phosphotransferase (PTS) systems, corroborated by API 50 CHL test results for carbohydrate metabolism profile. **Conclusions:** These findings enhance the comprehensive metabolic understanding of *L. fermentum* KUB-D18, supporting its industrial potential and applications in engineered probiotics.

## 1. Introduction

*Limosilactobacillus fermentum* KUB-D18 is a member of the *Lactobacillaceae* family, which is well known for producing lactic acid [1]. As a probiotic strain isolated from chicken intestines, *L. fermentum* KUB-D18 exhibits several probiotic properties [2]. These characteristics highlight strong potential roles in food fermentation and health promotion, such as modulating the gut microbiome, improving metabolic health, and enhancing immune function [3].

To gain a deeper understanding of *L. fermentum* KUB-D18, obtaining a complete genome sequence is one of the most effective tools for systematic level. Recently, various sequencing and annotation techniques further expand the possibilities for exploring the whole-genome sequence of *L. fermentum*. In the previous research reported by Phujumpa et al. [2], the short reads of *L. fermentum* KUB-D18 were obtained through Illumina NovaSeq 6000 platform sequencing and assembled into the first draft genome size of approximately 2.02 Mbps under 398 scaffolds with GC content of 51.7% and 2158 protein-encoding genes, which were then functionally annotated. The short-read genome of *L. fermentum* KUB-D18 reveals genes for folate biosynthesis, L-ascorbic acid metabolism, and bile salt hydrolase (BSH) activity. BSH enhances gastrointestinal survival by deconjugating bile acids [4]. It also lowers cholesterol by altering bile acid solubility, promoting excretion, and triggering cholesterol use for bile acid synthesis [4]. With the complete genome still unavailable, key gaps remain in understanding the metabolic functions linked to probiotic properties, including stress adaptation, antioxidant defense, as well as antimicrobial and anti-inflammatory activities [2].

Beyond metabolic gaps, critical information on sugar transport in *L. fermentum* KUB-D18 remains largely unknown. As sugars are essential nutrients for probiotic growth, a comprehensive analysis of its transportome is necessary [5]. The sugar transportome facilitates the uptake of all sugar molecules, such as glucose from the external environment into the cell, directly influencing the metabolic activity, growth, and overall functionality of probiotics [6]. By exploring all sugar transport-related genes of *L. fermentum* KUB-D18 and linking them to functional properties, e.g., lactic acid production, antioxidant activities, as well as other industrial perspectives, are basically required.

Recent advancements in sequencing technologies and bioinformatics tools have enabled integrating both short-read sequences data provided by Next-generation sequencing (NGS) technologies, e.g., Illumina, and long-read sequences data provided by third-generation sequencing (TGS) technologies, e.g., PacBio or Oxford Nanopore, to produce high-quality genomic assembly with resulting gaps reduction [7]. Bioinformatics tools, e.g., MaSuRCA, hybridSPAdes, and Unicycler, exemplified this advancement in more complete and accurate genome assemblies, thereby facilitating downstream genomic analysis [8,9]. This approach not only improves the resolution of complex genomic regions but also facilitates the identification of protein-encoding genes related to metabolic functions and sugar transports in probiotic bacteria [9,10,11].

This study, therefore, aimed to dissect metabolic functions and sugar transports using integrative genomic platform towards transportome analysis of probiotic *L. fermentum* KUB-D18. In brief, the integration of NGS by Illumina and TGS data by Oxford Nanopore was initially used for genome assembly of *L. fermentum* KUB-D18. Once a completed genome became available, the functional annotation and analysis was performed for investigating metabolic gene functions and sugar transports of *L. fermentum* KUB-D18 using protein and pathway databases. Finally, to evaluate potential probiotic properties within the context of carbon utilization, API 50 CHL test results for carbohydrate metabolism profile were performed.

Through this study, we gained a more comprehensive understanding of the metabolic basis of *L. fermentum* KUB-D18. The precise mapping of the metabolic and sugar transport functions also offers new possibilities for optimizing the strain’s growth conditions and enhancing its probiotic properties, thereby increasing potentials for industrial applications.

## 2. Materials and Methods

### 2.1. Culture Condition and Carbohydrate Metabolism Test of L. fermentum KUB-D18

*L. fermentum* strain KUB-D18, originally isolated from chicken intestine samples, was obtained from the stock culture of the Specialized Research Unit of Probiotics and Prebiotics for Health, Department of Biotechnology, Kasetsart University [12]. This strain was preserved at −20 °C in MRS medium (Difco Laboratories, Detroit, MI, USA) supplemented with 25% (*v*/*v*) glycerol. For experimental use, the culture was cultivated by two successive propagations in MRS medium with an initial pH of 6.5 ± 0.2 at 37 °C for 15 h each time. The carbohydrate metabolism test of this strain was evaluated by using the API 50 CHL kit according to the manufacturer’s protocol (Biomérieux, Marcy l’Etoile, France). In brief, the isolates strain was adjusted to the concentration of 2 Mc Farland standard in 2 mL API suspension and inoculated to API 50 CHL medium. The API 50 CHL medium was homogenized and aliquoted to API microtube strip. Prior to incubation, a layer of mineral oil was added, and the strip was incubated at 37 °C. The result was evaluated after 24 h of incubation and compared to the API database.

### 2.2. Genomic DNA Extraction Towards Sequencing of L. fermentum KUB-D18

*L. fermentum* KUB-D18 was cultured for 18 h on MRS agar plates supplemented with 0.05% L-cysteine. Genomic DNA was extracted by using the MagAttract HMW DNA Kit (Qiagen, Hilden, Germany) with slight modifications to the manufacturer’s protocol.

In brief, colonies were scraped from the agar surface and suspended in buffer P1 until the optical density at 600 nm (OD600) reached 0.5. To lyse the bacterial cells, 40 µL of lysozyme (200 mg/mL, Thermo Scientific, Waltham, MA, USA) was added to the suspension and gently mixed by tapping. The mixture was incubated at 37 °C for 1 h in a thermoshaker MATRIX Orbital (IKA, Staufen, Germany) with shaking at 600 rpm. Subsequently, 40 µL of proteinase K was added, mixed gently, and incubated at 56 °C with shaking at 600 rpm for 15 min.

To eliminate RNA contaminants, 4 µL of RNase A (100 mg/mL) was added, and the mixture was incubated statically at 37 °C for 10 min. The remaining steps followed the manufacturer’s standard protocol, with an additional DNA purification step using magnetic beads. The lysate was combined with magnetic bead suspension and a binding buffer, thoroughly mixed, and incubated to achieve purified DNA. The quality of genomic DNA was analyzed by NanoDrop Spectrophotometer (Thermo Scientific, Wilmington, DE, USA) and gel electrophoresis.

For genome sequencing, Oxford Nanopore Technology was performed to generate the integrative genome sequence of *L. fermentum* KUB-D18. For long-read Nanopore sequencing, the quality and adapter trimming of raw sequenced reads obtained was possessed by Nanoplot v1.38.0 [13] with a default parameter of a minimum length of 1000 base pairs (bps) and quality (Q) score of 10.

### 2.3. Integrative Genome Assembly and Functional Annotation of L. fermentum KUB-D18

**Figure 1:**
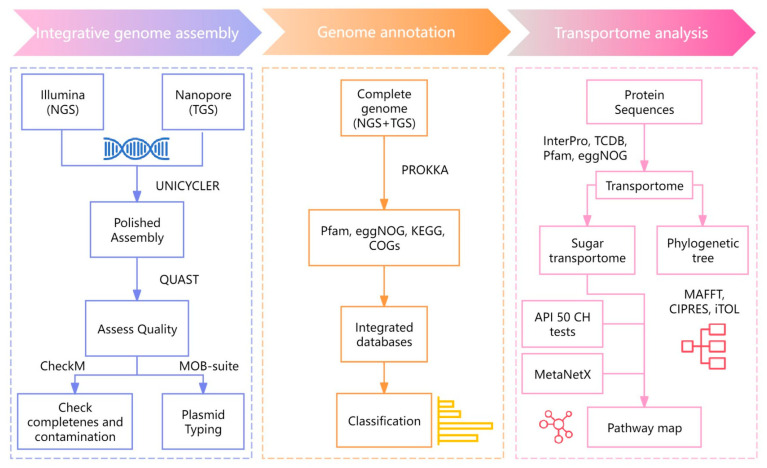
The workflow of integrative genome assembly and functional annotation of *L. fermentum* KUB-D18.

Integrative genome assembly was based on the short-read Illumina NovaSeq 6000 platform [2] and long-read Oxford Nanopore Technology. It was conducted using Unicycler v0.4.8 [14] and generating high-quality assembled sequences in FASTA format. The genome features of these sequences were assessed using the quality evaluation tool, i.e., QUAST v5.0.2 [15]. To verify the completeness and contamination of the assembled genome, CheckM v1.2.1 [16] was employed. Additionally, plasmid typing was performed using MOB-suite v3.1.5 [17] (Figure 1). All the sequences have been deposited in the Sequence Read Archive (www.ncbi.nlm.nih.gov/sra, accessed on 19 November 2024) under the accession no. PRJNA1187331 (SRA number: SRR31373359).

The genome of *L. fermentum* KUB-D18 was annotated by prokaryotic genome annotation (Prokka v1.14.6) using the default parameters and minimizing contig size to 1000 bps [18]. Functional annotation of the genes was performed using integrated protein and pathway databases, e.g., Pfam [19], KEGG [20], COGs, and eggNOG [21,22]. To further annotate core and strain-specific genes, comparative genes analysis was performed across related *L. fermentum* strains, e.g., 3872, CECT5716, and F-6 [23,24,25]. Those strains were firstly selected for comparison based on their well-documented probiotic characteristics and the availability of comparative genomic data for a functional analysis [26]. In addition, a list of targeted genes was also explored across 75 strains of *L. fermentum* genomes from IMG and NCBI databases.

### 2.4. Transportome Towards Metabolic Pathway Mapping of L. fermentum KUB-D18

Sequences were aligned against the Transporter Classification Database (TCDB) under thresholds of sequence identity of 40% and E-value of 1 × 10 ^−5^ [27]. Additionally, the Pfam, InterPro [28], and eggNOG databases were employed to screen for genes associated with transport functions. Then, Pfam, eggNOG, InterPro, and TCDB identifiers related to sugar transporters [29] were performed. Using the MAFFT v. 7.307 tool, multiple sequence alignment was performed for sugar transporters identification [30]. The phylogenetic tree was constructed using maximum likelihood analysis using RAxML–HPC BlackBox [31] through the CIPRES Science Gateway. The visualized phylogenetic tree was then illustrated using the online tool iTOL version 6 (https://itol.embl.de/, accessed on 26 December 2024) [32]. MetaNetX [33] was used to perform metabolic pathway mapping with functional annotation to further identify metabolic genes, enzymes and biochemical reactions to further propose metabolic functions illustrating diverse carbon utilization through various sugar transporters in *L. fermentum* KUB-D18.

## 3. Results and Discussion

### 3.1. Integrative Genomic Data Using NGS and TGS Towards Globally Annotated Results of L. fermentum KUB-D18

In this study, we conducted integrative genomics by assembling NGS (Illumina) [2] and TGS (Oxford Nanopore) data. As a result, the complete genome of *L. fermentum* KUB-D18 was successfully assembled into a single chromosomal contig, compared to the 398 scaffolds from the previous genome [2]. This greatly improved assembly continuity, minimized gaps and redundancies, and enhanced functional annotation (Table 1). As a result, the complete genome contains approximately 2.12 Mbps with GC content of 51.36%. Exploring annotation, it results in 2079 protein-encoding genes and 75 RNA-encoding genes.

In the context of enhancing functional annotation across updated multiple biological databases, the assigned protein functions were accordingly identified by COGs (1848 genes), KEGG (1222 genes), Pfam (1787 genes), and eggNOG (1849 genes) (Supplementary Tables S1–S4). After removing duplicated genes across integrated databases, the results were 1876 annotated genes (Table 1). This enables a comprehensive analysis of gene functions in the genome of the *L. fermentum* KUB-D18 and further links it to its probiotic functions.

**Figure 2:**
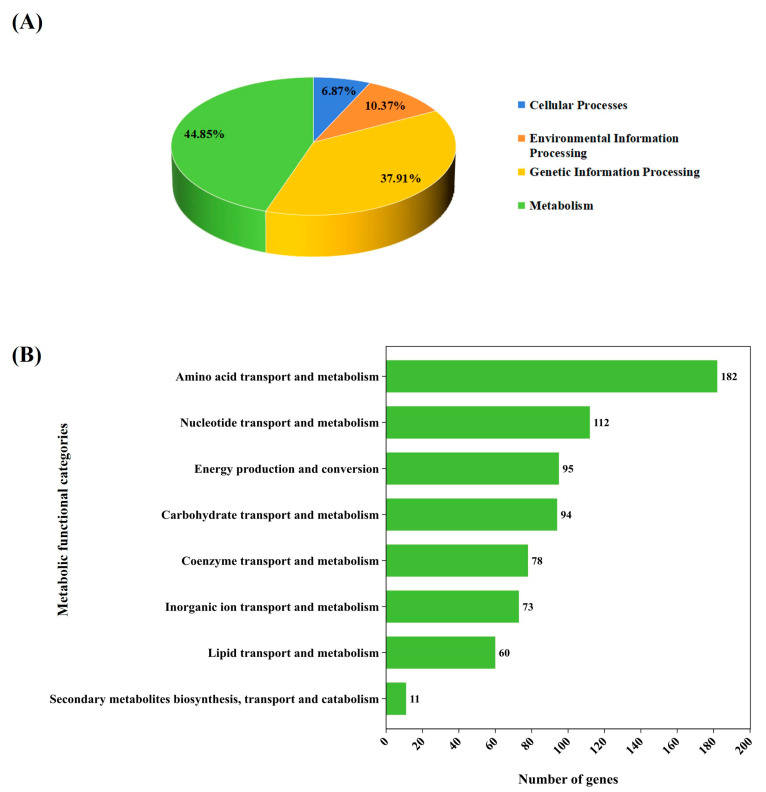
Classification of gene functions of *L. fermentum* KUB-D18 genome based on integrated databases. (**A**) The pie chart shows comparable percentage of gene functions distributed in four main functional categories. (**B**) The horizontal bar chart shows the comparable number of gene functions devoted to different metabolic functional categories.

Of the 1876 annotated genes, 1572 were classified into four main functional categories. The majority (705 genes, 44.85%) were involved in metabolism, followed by genetic information processing (596 genes, 37.91%), environmental information processing (163 genes, 10.37%), and cellular processes (108 genes, 6.87%) (Figure 2A). To further analyze the metabolic genes, we then categorized them into metabolic functional categories. The most abundant was involved in amino acid transport and metabolism (182 genes), followed by nucleotide transport and metabolism (112 genes), energy production and conversion (95 genes), carbohydrate transport and metabolism (94 genes), coenzyme transport and metabolism (78 genes), inorganic ion transport and metabolism (73 genes), lipid transport and metabolism (60 genes), and secondary metabolite biosynthesis, transport, and catabolism (11 genes) (Figure 2B).

Further, we identified abundance of genes in the histidine metabolic pathway (13 genes), lysine biosynthetic pathway (13 genes), purine metabolism (38 genes), and pyrimidine metabolism (24 genes). Additionally, 13 genes were associated with oxidative phosphorylation, 10 genes in nitrogen metabolism, 26 genes in glycolysis/gluconeogenesis, and 20 genes in pyruvate metabolism (Supplementary Table S5). Oxidative phosphorylation is a major ATP-producing pathway in many organisms, but heterofermentative lactobacilli, which do not favor oxygen, primarily rely on fermentation for energy production [34]. In the context of its probiotic function, these genes may support the strain’s survival in the less anaerobic part of the human gut. Additionally, they may contribute to enhancing oxidative phosphorylation in the host, potentially improving energy metabolism and nutrient utilization [35]. Further analysis of these metabolic genes and transport systems is needed to understand the strain-specific traits of *L. fermentum* KUB-D18.

### 3.2. Exploring the Metabolic Genes Revealing Core and Strain-Specific Characteristics of Probiotic L. fermentum KUB-D18

To investigate core and strain-specific genes in relation to probiotic properties of *L. fermentum* KUB-D18, 705 metabolic genes were initially explored for functions related to acid resistance, bile tolerance, antioxidant function, antimicrobial function, and anti-inflammatory property against the related *L. fermentum* strains (Materials and Methods). Regarding comparative genes analysis between KUB-D18 across these selected strains, a total of 50 core genes were identified, namely acid resistance, bile tolerance and metabolic adaptability (16 genes), antioxidant functions (21 genes), antimicrobial functions (5 genes), and anti-inflammatory properties (8 genes) (Table 2, Supplementary Table S6). Promisingly, *L. fermentum* KUB-D18 displayed 12 strain-specific genes related to metabolic adaptability (11 genes), and antioxidant functions (1 gene), as detailed in Table 2.

Comparative genes analysis revealed that *L. fermentum* KUB-D18 exhibits acid resistance and bile tolerance properties, supported by the presence of 8 core *atp* genes, i.e., *atpA* gene (GH00514), *atpB* (GH00510), *atpC* (GH00517), *atpD* (GH00516), *atpE* (GH00511), *atpF* (GH00512), *atpG* (GH00515), and *atpH* (GH00513). The *atp* genes in *L. fermentum* strains suggest an increased ATP production capacity, which likely supports its survival under acidic stress condition [36,37]. Moreover, six core *ldh* genes (GH01323, GH01664, GH01127, GH00286, GH00416, GH00725) encoding L-lactate dehydrogenase (EC: 1.1.1.27), which is the key enzyme of fermentation by lactic acid bacteria, and two core *bsh* genes (GH00033, GH01152) encoding bile salt hydrolase (EC: 3.5.1.24) were identified in *L. fermentum* KUB-D18. These results are consistent with Phujumpa et al. [2]. Accordingly, the presence of multiple *ldh* gene copies may enhance enzyme activity, improving environmental adaptability and antioxidant potential [37,55]. Altogether, *L. fermentum* KUB-D18 demonstrates acid resistance and bile tolerance and cholesterol-lowering properties, making it a candidate for industrial probiotic yogurt production, which maintains viability and functionality under acidic conditions [56].

For *ula* genes, notable ones are *ulaA* (GH01065, GH02018) encoding ascorbate PTS system EIIC component, *ulaB* (GH01066, GH02017) encoding ascorbate PTS system EIIB component (EC: 2.7.1.194), *ulaC* (GH01064, GH02019) encoding ascorbate PTS system EIIA or EIIAB component (EC: 2.7.1.194), and others crucial for L-ascorbic acid metabolism, i.e., *ulaD* (GH02016) encoding 3-dehydro-L-gulonate-6-phosphate decarboxylase (EC: 4.1.1.85), *ulaE* (GH02030) encoding L-ribulose-5-phosphate 3-epimerase (EC: 5.1.3.22), *ulaF* (GH02029) encoding L-ribulose-5-phosphate 4-epimerase (EC: 5.1.3.4), and *ulaG* (GH01067, GH02020) encoding L-ascorbate 6-phosphate lactonase (EC: 3.1.1.-).

The complete *ula* gene cluster in *L. fermentum* KUB-D18, absent in other strains, highlights its potential for L-ascorbic acid metabolism. Compared with orthologous 75 strains, as shown in Supplementary Table S7, this is supported by the annotated function of the *ula*-encoded phosphotransferase transport system for ascorbic acid catabolism and metabolic capability [56]. This result suggests a potential role in the high metabolic capability of *L. fermentum* KUB-D18, as seen in high copy numbers of genes, e.g., genes encoding the ascorbate PTS system [40].

Based on our findings, *L. fermentum* KUB-D18 harbors genes associated with antioxidant functions, such as folate (vitamin B9) [41,42]. The identification of comparative genes revealed eight core *fol* genes involved in folate biosynthesis, including *folA* (GH01227), *folB* (GH01551), *folC* (GH00605, GH01548), *folD* (GH01469), *folK* (GH01550), *folP* (GH01546), and *folE* (GH01549) (Supplementary Table S6) [2]. The folate regulates homocysteine levels, supporting cysteine and glutathione synthesis to combat oxidative stress [57]. Functional genes analysis identified three core glutamate-cysteine ligase (*gshA*) genes (EC: 6.3.2.2) (GH00749, GH01002, GH01072) essential for glutathione synthesis [43,58], along with glutathione reductase genes, i.e., two core genes (GH01873, GH02130) and a strain-specific gene (GH01400) [44]. A core gene (GH00480) encoding NADH-dependent peroxiredoxin subunit C and seven thioredoxin system genes (*trxA*: GH00635, GH01874, GH01968, GH02124; *trxB*: GH00410, GH00481, GH02127) further support redox balance and oxidative damage repair in *L. fermentum* KUB-D18 [45,46,47].

Furthermore, we also identified three core genes related to exopolysaccharide (EPS) biosynthesis in *L. fermentum* KUB-D18, including *epsA* (GH00095, GH01646) encoding protein tyrosine kinase modulator and *epsB* (GH00096) encoding protein tyrosine kinase. EPS forms a dense extracellular matrix that serves as a protective barrier, preventing pathogenic adhesion and biofilm formation [50,59]. Antimicrobial functions in *L. fermentum* KUB-D18 were linked to two core genes involved in the acetic acid biosynthetic pathway: *GH00336* encoding acetaldehyde dehydrogenase/alcohol dehydrogenase (EC: 1.2.1.10; 1.1.1.1) and *GH00451* encoding phosphate acetyltransferase (EC: 2.3.1.8) [48,49]. Acetic acid is a primary short-chain fatty acid (SCFA); this suggests that it might be a key role in maintaining energy balance and metabolic homeostasis in *L. fermentum* KUB-D18 [60].

Considering anti-inflammatory properties, we identified five core genes related to histidine metabolism in *L. fermentum* KUB-D18: *hisF* (GH00861, GH00869), *hisH* (GH00859, GH00867), and *hisJ* (GH00862), encoding essential enzymes, e.g., imidazole glycerol-phosphate synthase (EC: 4.3.2.10) and histidinol-phosphatase (EC: 3.1.3.15) involved in the production of histidinol phosphate, a key intermediate in histamine biosynthesis [51]. Histidine-derived histamine modulates immune cell activity, helping maintain immune balance and exerting anti-inflammatory effects [61,62]. Previous studies have shown that *his* genes exert anti-inflammatory effects supported by animal model experiments [62] by regulating pro-inflammatory mediators, such as tumor necrosis factor (Tnf)-α and interleukin (Il)-6.

Additionally, genes linked to glutamate and glutamine metabolism, including GH01482 (glutamine synthetase, EC: 6.3.1.2) and GH01059, GH01060 (glutamate:GABA antiporter/glutamate decarboxylase, EC: 4.1.1.15), indicate the presence of a metabolic pathway for GABA synthesis, suggesting roles in stress tolerance, anti-inflammatory effects, and gut–brain axis modulation [52,53,63,64,65]. Previous studies have shown that *L. fermentum* KUB-D18 exhibits high resistance in an in vitro gastrointestinal model, surviving stomach and small intestinal conditions while modulating the gut microbiota of overweight individuals in a colon fermentation model. This adaptability suggests its ability to adjust to metabolic activity in response to environmental changes. This metabolic flexibility was observed in conjunction with *L. reuteri* KUB-AC5, indicating a synergistic effect rather than an isolated trait of *L. fermentum* KUB-D18 alone [66].

Altogether, these findings not only highlight *L. fermentum* KUB-D18′s probiotic properties but also provide a foundation for use in functional foods and probiotic supplements. Since the probiotic functionality often depends on their sugar uptake and metabolism, which provide energy to cells, drive key metabolic pathways, and support the probiotic functions, further studies on of *L. fermentum* KUB-D18′s metabolic transports are essential for understanding probiotic effects.

### 3.3. Identification of Metabolic Transports of L. fermentum KUB-D18 Using Transportome Analysis

**Figure 3:**
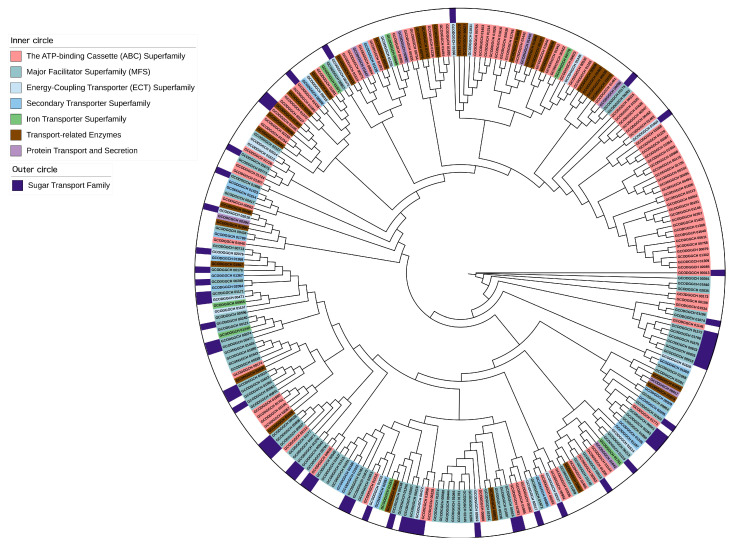
The maximum likelihood phylogenetic tree of metabolic transporter genes distributing in different transporter categories of *L. fermentum* KUB-D18. The phylogenetic tree was initially produced using RAxML–HPC BlackBox through the CIPRES Science Gateway with default parameters and then visualized by the online tool iTOL version 6 (https://itol.embl.de/, accessed on 26 December 2024).

Apart from the metabolic genes relevant to metabolism, how metabolic genes are transported across the cellular membrane or between compartments remains unknown in *L. fermentum* KUB-D18. To explore the metabolic transporters in *L. fermentum* KUB-D18, transportome analysis was performed on 2079 protein-encoding genes, with 14.76% (307 genes) encoding transmembrane proteins. These were annotated using TCDB (155 genes), Pfam (127 genes), eggNOG (58 genes), and InterPro (44 genes) (Table 3). Across integrated databases, 259 genes were related to metabolic transports (Supplementary Table S8). A maximum likelihood tree classified these into seven transporters’ categories: ATP-binding cassette (ABC) superfamily (97 genes, 37.5%), major facilitator superfamily (MFS) (82 genes, 31.7%), transport-related enzymes (30 genes, 11.6%), energy-coupling transporter (ECT) superfamily (20 genes, 7.7%), secondary transporter superfamily (16 genes, 6.2%), protein transport and secretion (7 genes, 2.7%), and iron transporter superfamily (7 genes, 2.7%) (Figure 3).

In lactic acid bacteria, the sugar transporters were promisingly required to study in *L. fermentum*, and thus the term of sugar transportome has been raised to represent whole sugar transporters, which were investigated through advanced bioinformatics and next generation sequencing technology [29,67].

#### 3.3.1. Sugar Transportome Analysis Towards Alternative Carbon Substrate Utilization of *L. fermentum* KUB-D18

In this study, a number of sugar transporters (57 of 259 metabolic transporter genes) were identified in the *L. fermentum* KUB-D18 genome (Table 4). They were classified into three groups, including secondary carriers (43 genes), e.g., the Major Facilitator Superfamily (MFS) and drug/metabolite transporter (DMT) superfamilies (Table 5), PTS systems (11 genes), and ABC carriers (3 genes) (Supplementary Table S9). These transporters play key roles in carbohydrate metabolism. MFS transporters facilitate the passive diffusion of sugars along their concentration gradient [68], while the PTS family actively couples sugar entry with phosphorylation, altering sugar structures [69]. ABC transporters, energy-dependent systems, enable sugar uptake via ATP hydrolysis, typically with high substrate specificity. As illustrated in Figure 3, phylogenetic analysis also revealed that genes from the same family did not cluster in a single branch, suggesting evolutionary divergence. This diversity is likely due to factors such as varying substrate specificity [70] and/or environmental adaptations [71]. These findings highlight the complexity and evolutionary diversity of sugar transporter functions in *L. fermentum* KUB-D18.

#### 3.3.2. Probing Carbon Utilization in *L. fermentum* KUB-D18

**Figure 4:**
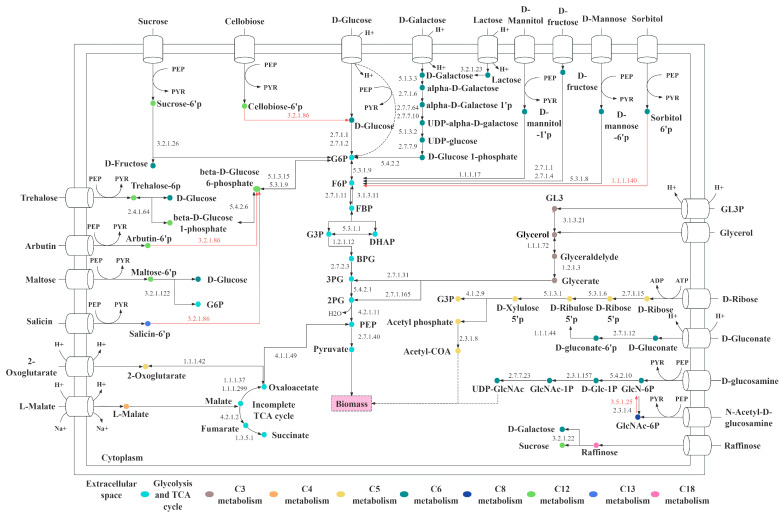
The proposed core metabolic functions illustrate diverse carbon utilization through various sugar transporters in *L. fermentum* KUB-D18. The red line indicates the missing gene encoding the enzyme. Abbreviated metabolite names are as follows: G6P, glucose 6-phosphate; F6P, β-fructose 6-phosphate; G3P, glyceraldehyde 3-phosphate; DHAP, dihydroxyacetone phosphate; GL3P, glycerol 3-phosphate; PEP, phosphoenolpyruvate; PYR, pyruvate; 2PG, 2-phospho-glycerate; 3PG, 3-phospho-glycerate.

Based on the sugar transportome analysis, hereby we mapped the 57 sugar transporter genes in the metabolism of *L. fermentum* KUB-D18. As illustrated in Figure 4, the transport genes of the following carbon as substrate including C3 (glycerol-3-phosphate, glycerol), C4 (L-malate), C5 (2-oxoglutarate, D-ribose), C6 (D-glucose, D-fructose, D-galactose, D-glucosamine, D-gluconate, D-mannose, D-mannitol, D-sorbitol, lactose), C8 (*n*-acetyl-D-glucosamine), C12 (trehalose, arbutin, maltose, sucrose, cellobiose), C13 (salicin), and C18 (raffinose) were mapped.

It is worth noting that lactose and raffinose are key carbon sources for probiotics, offering distinct benefits beyond industrial staples, like glucose, sucrose, or maltose. Lactose utilization helps prevent lactose allergies and supports lactose-intolerant individuals, making *L. fermentum* KUB-D18 a promising candidate for probiotic products targeting this demographic, with added industrial potential [72]. Raffinose promotes beneficial gut bacteria, improves gut health, and it is low-calorie and non-digestible by human, enhancing the health benefits and market value of probiotic products [73]. Understanding these pathways highlights *L. fermentum* KUB-D18′s genetic basis for probiotic properties, supporting its optimization for functional foods and health-related applications. Comparing with other strains, *L. fermentum* CECT5716, for example, was experimentally validated to utilize similar carbon sources, e.g., glucose, maltose, lactose, and raffinose for growth [26]. In addition to specific carbon sources used, a presence of PTS system identified in genome could guide the ability to uptake carbon substrates.

Interestingly, 11 out of 57 genes were also related to the PTS system (Figure 4), revealing its critical role in carbon metabolism. Many carbon substrates, such as sucrose, D-mannose, and maltose, enter the cell via the PTS pathway. This system is specialized with phosphorylate substrates during transport, enabling their direct entry into subsequent metabolic pathways. This process enhances the efficiency of carbon substrate utilization [74]. Furthermore, the PTS system regulates substrate priority, allowing cells to preferentially metabolize glucose while inhibiting the utilization of other carbon sources. This mechanism optimizes metabolic efficiency under resource-limited conditions, maximizing microbial growth rates [75]. Carbon utilization analysis in *L. fermentum* KUB-D18 revealed potential transport pathways for glycerol, D-mannitol, and trehalose (Figure 4), while the strain was unable to metabolize arbutin, salicin, cellobiose, sorbitol, or glucosamine, as confirmed by API 50 CH tests (listed in Supplementary Table S10). Most of the carbon sources that cannot be utilized may be due to the absence of metabolic genes or the fact that the associated genes have not been linked to the metabolism of those carbon sources. In addition, it might be because of enzyme preference and substrate specificity [76].

It is worth noting that although transporter genes for sorbitol have been identified, an enzyme required to convert sorbitol 6-phosphate into fructose-6-phosphate is missing (Figure 4). Thus, strain KUB-D18 cannot grow using sorbitol as a specific source. These findings highlight its adaptation and growth characteristics. Functional studies of metabolic transporters together with metabolic genes are crucial for engineering *L. fermentum* KUB-D18 to improve interactions with gut microbiota, including nutrient acquisition (e.g., sugars) and metabolism, adhesion to gut surfaces, as well as key in maintaining gut homeostasis and preventing dysbiosis [77,78].

## 4. Conclusions

A complete genome of *L. fermentum* KUB-D18 was successfully assembled using integrative next-generation and third-generation sequencing. Key genes linked to probiotic properties, for instances acid resistance, bile tolerance, antioxidant functions, and anti-inflammatory properties, were identified. This supports the annotation and analysis of a prior result from *L. fermentum* KUB-D18 [2]. The sugar transportome analysis revealed sugar transporter genes, indicating diverse carbon utilization and PTS systems, consistent with API 50 CHL test results. This study provides valuable insight into the genotype–phenotype relationship of *L. fermentum* KUB-D18, supporting its potential for industrial and engineered probiotic applications. Future research should focus on comprehensive in vitro and in vivo investigations to validate the probiotic potential of *L. fermentum* KUB-D18, particularly its metabolic function, activity and impact on gut health, e.g., L-ascorbic acid metabolism through physiological studies, transcriptomics, proteomics or metabolomics. Additionally, engineering *L. fermentum* KUB-D18 to enhance the production of beneficial metabolites could improve its potential for treating inflammatory bowel diseases and balancing gut microbiota. Finally, exploring *L. fermentum* KUB-D18′s application in functional foods and probiotics provides valuable insights into optimizing gut health and antioxidant capacity. Ultimately, this research contributes to the development of next-generation probiotics with enhanced therapeutic properties, paving the way for novel dietary interventions that promote long-term gut health and overall well-being.

## Figures and Tables

**Table 1:** Comparison of genomic characteristics of *L. fermentum* KUB-D18 in different studies.

Genomic Characteristics	Phujumpa et al. [2]	This Study
Genome sequence (bps)	2,016,883	2,122,415
GC content (%)	51.70	51.36
No. of protein-encoding genes	2158	2079
No. of RNA-encoding genes	65	75
Scaffolds	398	1
DB-based protein functional annotation		
KEGG	961	1222
Pfam	-	1787
EggNOG	-	1849
COGs	-	1848
Integrated databases	1810	1876

Note: DB stands for database.

**Table 2:** List of core and strain-specific genes related to probiotic functions of *L. fermentum* KUB-D18 in comparative analysis with other relevant strains.

Features	Properties	Symbol	Description	Abbreviated Gene ID	Strains				Refs.
					KUB-D18	3872	CECT5716	F-6	
Acid resistance, bile tolerance, metabolic capability	Acid tolerance	*Atp*	ATPase (3.6.1.3)	GH00510	+	+	+	+	[36,37]
				GH00511	+	+	+	+	
				GH00512	+	+	+	+	
				GH00513	+	+	+	+	
				GH00514	+	+	+	+	
				GH00515	+	+	+	+	
				GH00516	+	+	+	+	
				GH00517	+	+	+	+	
		*Ldh*	L-lactate dehydrogenase (1.1.1.27)	GH01127	+	+	+	+	[37]
				GH01323	+	+	+	+	
				GH01664	+	+	+	+	
				GH00286	+	+	+	+	
				GH00416	+	+	+	+	
				GH00725	+	+	+	+	
	Bile salt tolerance	*Bsh*	Choloylglycine hydrolase (3.5.1.24)	GH00033	+	+	+	+	[38,39]
				GH01152	+	+	+	+	
	Metabolic capability	*Ula*	L-ascorbic acid metabolism (2.7.1.194 3.1.1.- 4.1.1.85 5.1.3.4 5.1.3.22)	GH01064	+ *	−	−	−	[2,40]
				GH01065	+ *	−	−	−	
				GH01066	+ *	−	−	−	
				GH01067	+ *	−	−	−	
				GH02016	+ *	−	−	−	
				GH02017	+ *	−	−	−	
				GH02018	+ *	−	−	−	
				GH02019	+ *	−	−	−	
				GH02020	+ *	−	−	−	
				GH02029	+ *	−	−	−	
				GH02030	+ *	−	−	−	
Antioxidant function	Metabolism of antioxidant molecules	*Fol*	Folate biosynthesis (6.3.2.12 6.3.2.17 1.5.1.3 1.5.1.5 3.5.4.9 2.5.1.15 3.5.4.16 2.7.6.3 4.1.2.25 5.1.99.8 1.13.11.81)	GH00605	+	+	+	+	[41,42]
				GH01227	+	+	+	+	
				GH01469	+	+	+	+	
				GH01546	+	+	+	+	
				GH01548	+	+	+	+	
				GH01549	+	+	+	+	
				GH01550	+	+	+	+	
				GH01551	+	+	+	+	
		*gshA*	Glutamate--cysteine ligase (6.3.2.2)	GH00749	+	+	+	+	[43]
				GH01002	+	+	+	+	
				GH01072	+	+	+	+	
		*Gor*	Glutathione reductase (NADPH) (1.8.1.7)	GH01400	+ *	−	−	−	[43,44]
				GH01873	+	+	+	+	
				GH02130	+	+	+	+	
		*ahpC*	NADH-dependent peroxiredoxin subunit C (1.11.1.26)	GH00480	+	+	+	+	[45]
	Repairing oxidized proteins	*Trx*	Thioredoxin; thioredoxin reductase (1.8.1.9)	GH00635	+	+	+	+	[46,47]
				GH01874	+	+	+	+	
				GH01968	+	+	+	+	
				GH02124	+	+	+	+	
				GH00410	+	+	+	+	
				GH00481	+	+	+	+	
				GH02127	+	+	+	+	
Anti-microbial substances	Organic acid synthesis	*adhE*	Acetaldehyde dehydrogenase /alcohol dehydrogenase (1.2.1.10 1.1.1.1)	GH00336	+	+	+	+	[48]
		*Pta*	Phosphate acetyltransferase (2.3.1.8)	GH00451	+	+	+	+	[49]
	Extracellular polymers (EPS)	*Eps*	Protein-tyrosine kinase (2.7.10.3)	GH00095	+	+	+	+	[50]
				GH00096	+	+	+	+	
				GH01646	+	+	+	+	
Anti-inflammatory substances	Anti-inflammatory regulatory factors	*hisJ*, *hisF*, *hisH*	Histidine metabolism (4.3.2.10 3.1.3.15)	GH00862	+	−	+	+	[51]
				GH00861	+	−	+	+	
				GH00869	+	−	+	+	
				GH00859	+	−	+	+	
				GH00867	+	−	+	+	
		*Gad*	Glutamate:GABA antiporter; glutamate decarboxylase (4.1.1.15)	GH01059	+	−	−	+	[52,53]
				GH01060	+	−	−	+	
		*Glu*	Glutamine synthetase (6.3.1.2)	GH01482	+	+	+	+	[54]

Note: Comparative genes analysis under thresholds (%Identity > 40% and E-value < 1 × 10 ^−5^). Full details can be found in Supplementary Table S6. (*) indicates a strain-specific gene of KUB-D18 when compared to other strains (Supplementary Table S6), (+) indicates gene presence, (−) indicates gene absence.

**Table 3:** List of metabolic transporter genes across integrated protein and transporter databases.

Protein/Transporter DB	Metabolic Transporter Genes
TCDB	155
Pfam	127
eggNOG	58
InterPro	44
Integrated DB	259 *

Note: * a set of metabolic transporter genes sourced from Integrated DB (TCDB, Pfam, eggNOG, InterPro), with duplicates removed.

**Table 4:** List of sugar transporter genes across integrated protein and transporter databases.

Protein/Transporter DB	Sugar Transporter Genes
TCDB	10
Pfam	57
InterPro	26
Integrated DB	57

**Table 5:** List of sugar transporter genes categorized into secondary carriers, PTS systems, and ABC carriers.

COG/Pfam	Description	Sugar Transporter Genes
Secondary Carriers		
COG0580	Glycerol uptake facilitator and related permeases (Major Intrinsic Protein Family)	1
COG0697	Permeases of the drug/metabolite transporter (DMT) superfamily	6
COG2814	Arabinose efflux permease	2
Pfam00083	Sugar transporter (MFS)	11
Pfam06800	Sugar transport proteins	2
Pfam07690	Major Facilitator Superfamily (MFS_1)	21
PTS Systems		
COG1762	PTS mannitol/fructose-specific IIA domain	2
COG2893	PTS mannose/fructose-specific component IIA	1
COG3715	PTS mannose/fructose/*n*-acetylgalactosamine specific component IIC	1
COG3716	PTS mannose/fructose/*n*-acetylgalactosamine specific component IID	1
COG3775	PTS galactitol-specific IIC component	1
Pfam00358	PTS_EIIA_1	2
Pfam02302	PTS_IIB: Lactose/Cellobiose specific	2
Pfam02378	PTS_EIIC	1
ABC Carriers		
Pfam01061	ABC2_membrane (transport of carbohydrates)	1
Pfam01547	SBP_bac_1 (bacterial extracellular solute-binding protein, e.g., maltose)	1
Pfam02653	BPD_transp_2 (branched chain amino acid transport system, permease component; family also contains a galactose and ribose transport system)	1

## Data Availability

All the sequences have been deposited in the Sequence Read Archive (https://www.ncbi.nlm.nih.gov/sra, accessed on 19 November 2024) under the accession no. PRJNA1187331 (SRA number: SRR31373359).

## Supplementary Materials

The following supporting information can be downloaded at https://www.mdpi.com/article/10.3390/genes16030348/s1, Table S1: The COG category classification of *L. fermentum* KUB-D18. Table S2: The KEGG annotation of *L. fermentum* KUB-D18. Table S3: The Pfam annotation of *L. fermentum* KUB-D18. Table S4: The eggNOG annotation of *L. fermentum* KUB-D18. Table S5: The gene list for the primary subcategories within the main functional categories of *L. fermentum* KUB-D18. Table S6: List of core and strain-specific genes related to probiotic functions of *L. fermentum* KUB-D18 in comparative analysis with other strains. Table S7: List of core and strain-specific genes-related to ula gene cluster of *L. fermentum* KUB-D18 in comparative analysis with other strains. Table S8: The genes related to transmembrane protein of *L. fermentum* KUB-D18. Table S9: The sugar transport related genes of *L. fermentum* KUB-D18. Table S10: The API 50 CHL carbohydrate metabolism test result of *L. fermentum* KUB-D18.
